# Perspectives on chronic granulomatous disease: results of a clinician survey

**DOI:** 10.3389/fimmu.2026.1731304

**Published:** 2026-07-16

**Authors:** Joao Pedro Matias Lopes, Ben Z. Katz, Niraj C. Patel, Kelli W. Williams

**Affiliations:** 1 Division of Pediatric Allergy, Immunology, and Rheumatology, University Hospitals Rainbow Babies & Children’s Hospital, Case Western Reserve University, Cleveland, OH, United States; 2 Division of Infectious Diseases, Ann & Robert H. Lurie Children’s Hospital of Chicago, Chicago, IL, United States; 3 Department of Pediatrics, Northwestern University Feinberg School of Medicine, Chicago, IL, United States; 4 Division of Allergy and Immunology, Department of Pediatrics, Duke University, Durham, NC, United States; 5 Division of Pediatric Pulmonology, Allergy, and Immunology, Medical University of South Carolina, Charleston, SC, United States

**Keywords:** carriers of X-linked CGD, chronic granulomatous disease, clinical practice survey, diagnostic trends, expert perspectives, inborn errors of immunity, primary immune deficiency, treatment decisions

## Abstract

**Background:**

Chronic granulomatous disease (CGD) is a primary immune deficiency characterized by neutrophils with an insufficient oxidative burst. Symptoms include infections and inflammatory manifestations affecting the lungs, lymph nodes, liver, bone, soft tissue, and other organs. Diagnostic and treatment approaches may vary between patients with X-linked CGD and patients with autosomal recessive CGD, depending on their age at diagnosis and the type and severity of their symptoms. Female carriers of X-linked CGD may also manifest symptoms of CGD that require tailored treatment and monitoring over time, though there is little consensus on management strategies for this population. The aim of this study was to measure perceptions among US-based clinicians on current diagnostic, treatment, and monitoring practices for patients with CGD, including variance in approaches across CGD subtypes.

**Methods:**

A 25-item survey was distributed to clinicians in various disciplines with experience in CGD care. Questions probed clinicians’ overall approaches to CGD diagnosis, barriers to CGD care, preferred treatments, and factors influencing treatment decisions. When relevant, results were stratified to show differences in clinical approaches for male patients with X-linked CGD, patients with autosomal recessive CGD, and symptomatic female carriers of X-linked CGD.

**Result:**

Clinicians ranked the presence of CGD-associated pathogens as the factor most frequently leading to CGD identification, over the presence of minor or major infections, occurrence of noninfectious manifestations, and family history. Specialty referrals were likely to lead to a CGD diagnosis only sometimes or seldom. Infection severity was ranked as the top factor influencing CGD treatment recommendations across all CGD subtypes, although actual reported treatment decisions varied substantially for male patients with X-linked CGD and patients with autosomal recessive CGD vs. symptomatic female carriers of X-linked CGD.

**Conclusion:**

This survey is the first multidisciplinary US survey focused on clinician perceptions of current CGD care practices, including management of symptomatic female carriers of X-linked CGD. The results highlight the need for greater evidence-based clinical consensus in CGD diagnostic and treatment practices, particularly for symptomatic female carriers of X-linked CGD, and the importance of amplifying clinical suspicion for CGD among specialty clinicians to increase timely CGD diagnosis.

## 1 Introduction

Chronic granulomatous disease (CGD) is a rare, heterogeneous primary immune deficiency caused by a defect in 1 of 6 genes that encode for or permit assembly of the subunits of nicotinamide adenine dinucleotide phosphate oxidase (NADPH). These genetic defects result in a deficient neutrophil oxidative burst, increasing an individual’s risk for bacterial or fungal infections, typically occurring in the lungs, lymph nodes, liver, bone, or soft tissue (1). A wide range of noninfectious, inflammatory manifestations affecting the gastrointestinal tract, lungs, skin, and other organs may also occur (2). Most patients are diagnosed before 5 years of age; however, CGD may present anytime from infancy to late adulthood (1).

CGD typically follows a pattern of either X-linked or autosomal recessive inheritance, though *de novo* cases also occur (1). X-linked CGD (XL-CGD) accounts for approximately two-thirds of CGD cases in the US (3, 4). Male individuals with XL-CGD are typically diagnosed earlier in life and present with more severe manifestations, including serious bacterial and fungal infections (1, 5). In contrast, autosomal recessive CGD (AR-CGD) can be more challenging to identify due to its later onset, often outside of early childhood, and less severe clinical phenotype (1, 6, 7). An additional distinct, but overlooked, CGD subtype is that of the female carrier of XL-CGD who may be asymptomatic or symptomatic with clinical manifestations of CGD due to skewed X-chromosome inactivation. Autoimmune or inflammatory manifestations in this population may include discoid lupus erythematosus, oral ulcers, inflammatory bowel disease, and polyarthritis, among others (8). Carriers of XL-CGD with skewed lyonization are also susceptible to CGD-associated infections, which may warrant prophylactic antimicrobials (9).

The estimated incidence of CGD in the US is 1 case per 200,000 live births (5), although recent studies suggest the incidence may be higher (4). The dihydrorhodamine (DHR) assay is considered the gold standard test to establish a diagnosis of CGD (10, 11); however, CGD diagnosis is not always straightforward, given the variability in symptoms and age of presentation across subtypes. While specialists such as allergist-immunologists, infectious disease clinicians, pulmonologists, rheumatologists, and hematologists are critical in identifying early signs of CGD, CGD awareness among potential referring physicians is low owing to the rarity of the disease and overlapping symptoms that are also seen in immunocompetent individuals (3, 12–14). Misdiagnosis due to factors such as unusual presentation, uncommon pathogen involvement, mild phenotype, and lack of clinical suspicion for CGD have been reported (12).

The foundations of CGD management are prompt and aggressive diagnosis and treatment of acute infections, implementation of daily antibacterial and antifungal prophylaxis, immune modulation with interferon gamma-1b, ongoing monitoring, and avoidance of environmental factors that increase the risk of exposure to harmful bacterial microbes or fungal spores (1, 15). Collaboration among specialists to manage multiorgan complications is critical (2). Hematopoietic stem cell transplantation (HSCT) has curative potential and good outcomes when performed in patients of young age with matched donors and in patients without end organ damage (1). Next-generation therapeutics, such as gene therapy and gene editing, are also being investigated to treat CGD (16).

Though CGD treatment options are well defined, multiple clinical challenges related to management decisions currently exist; for example, determining the best treatment course (i.e., long-term medical management or HSCT), optimizing timing and type of disease surveillance, and balancing use of immunosuppressive treatment for noninfectious manifestations in a condition with an increased underlying risk for infection (1, 2, 17, 18). Furthermore, there are no disease-specific, evidence-graded CGD management guidelines from any US or European societies (1, 2, 19). Without standardized guidelines, clinicians prioritize a mix of learnings from the available literature and their real-world experience to inform CGD management. Psychosocial factors, such as mental health, health-care access, medication nonadherence, and social determinants of health (SDOH), may also impact treatment decisions and overall patient well-being (1, 3, 19, 20), further complicating the global management picture.

To assess current real-world practices and perspectives, a clinical practice survey was undertaken among US clinicians who care for patients with CGD. Here we summarize the survey results, including findings related to diagnostic factors, treatment decisions across CGD subtypes, barriers to effective CGD care, and potential areas of discord among US-based clinicians.

## 2 Materials and methods

### 2.1 Survey instrument

A 25-item survey was developed and included multiple choice, ranking, and matrix-style questions. These questions focused on CGD diagnosis, treatment, and barriers to care for male patients with XL-CGD, patients with AR-CGD, and symptomatic and asymptomatic female carriers of XL-CGD. Demographic information, including respondents’ primary medical specialty, years in practice, age of patients seen, and number and type of patients with CGD, was also collected. The text of the survey is available in the **Supplementary Materials**.

### 2.2 Survey sampling and data collection

A purposive sampling approach was used to identify >350 clinicians with potential experience in CGD care. Potential participants were identified based on the authors’ awareness of health-care professionals who diagnose or manage CGD or related inborn errors of immunity, including pediatric and adult immunologists, infectious disease specialists, and transplant physicians. The study sponsor also assisted with identifying additional clinicians who met these criteria. Survey invitations were sent to all identified clinicians for whom contact information was available, without regard to their perspectives on CGD management, and responses were collected anonymously. To be eligible to complete the survey, respondents were required to self-attest current or prior clinical experience caring for patients with CGD and to be practicing within the US. Responses from clinicians practicing outside the US were excluded to minimize geographic variability that could confound interpretation (eg, health-system factors, relative prevalence of XL-CGD vs AR-CGD, etc).

### 2.3 Data analysis

Descriptive statistics were calculated for each survey item (frequencies for categorical variables; means, standard deviations, medians, and ranges for continuous variables). Friedman rank sum testing was used to measure differences in matrix question responses and Wilcoxon rank sum testing was used to analyze responses to ranked-answer questions. Incomplete surveys were allowed and were included in the data analyses. All calculations were performed manually in Microsoft Excel and using XLSTAT (Lumivero; Denver, CO).

## 3 Results

### 3.1 Sample demographics

A total of 43 US-based clinicians completed the survey, and 36 responses were included in the data analyses. The excluded responses comprised 6 who chose not to complete the survey beyond the screening questions and 1 respondent who did not have direct CGD patient care experience.

Twenty-seven respondents provided demographic information (**Table 1**). Of these respondents, 59% (n=16) specialize in allergy/immunology, 22% (n=6) specialize in infectious disease, 11% (n=3) specialize in hematology/oncology, and 7% (n=2) belong to other specialties. 44% (n=12) have ≥21 years of experience in their current specialty, 37% (n=10) have 11 to 20 years of experience, and 19% (n=5) have 1 to 10 years of experience.

**Table 1:** Survey respondent demographics (n=27)^*^.

Category	Response, n (%)
Primary medical specialty	
Allergy/Immunology	16 (59.3)
Infectious disease	6 (22.2)
Hematology/Oncology	3 (11.1)
Other^†^	2 (7.4)
Duration in current medical specialty	
0 to 10 years	5 (18.5)
11 to 20 years	10 (37.0)
≥21 years	12 (44.4)
Patient population age group	
Both adult and pediatric patients	17 (62.9)
Mainly pediatric (aged ≤17 years)	8 (29.6)
Mainly adult (aged ≥18 years)	2 (7.4)
Number of patients per CGD subtype	
Male patients with XL-CGD	
1 to 10	13 (48.1)
11 to 20	6 (22.2)
≥21	7 (25.9)
None	1 (3.7)
Patients with AR-CGD	
1 to 10	21 (77.7)
11 to 20	4 (14.8)
≥21	0 (0)
None	2 (7.4)
Symptomatic female carriers of XL-CGD	
1 to 10	21 (77.7)
11 to 20	0 (0)
≥21	1 (3.7)
None	5 (18.5)
Asymptomatic female carriers of XL-CGD with low %DHR+	
1 to 10	12 (44.4)
11 to 20	3 (11.1)
≥21	1 (3.7)
None	11 (40.7)

^*^ Of respondents, 9 did not provide demographic information and are therefore not included in this table.

^†^ Responses included diagnostic immunology and immune deficiency/bone marrow transplant.

%DHR+, relative proportion of dihydrorhodamine fluorescence–positive neutrophils; AR-CGD, autosomal recessive chronic granulomatous disease; CGD, chronic granulomatous disease; XL-CGD, X-linked chronic granulomatous disease.

Most respondents reported having cared for <10 patients within each of the following CGD subtypes: male patients with XL-CGD, patients with AR-CGD, symptomatic female carriers of XL-CGD, and asymptomatic female carriers of XL-CGD with a low relative proportion of DHR fluorescence–positive neutrophils (%DHR+). The majority of respondents (63%) care for both adult and pediatric patients.

### 3.2 CGD identification

Respondents were asked to determine the frequency of CGD identification based on several clinical factors (i.e., major infections, minor infections, presence of CGD-associated pathogens, noninfectious manifestations, and CGD family history), irrespective of CGD subtype. The presence of CGD-associated pathogens (defined in the survey as *Staphylococcus aureus, Serratia marcescens, Burkholderia cepacia, Nocardia* species, *Aspergillus* species, and *Candida*) was ranked as the factor most frequently leading to CGD diagnosis, with 92% of clinicians agreeing that this clinical finding almost always (39%; n=14) or often (53%; n=19) leads to identification of CGD. CGD identified due to CGD-associated pathogens was reported significantly more frequently vs. minor infections treated in the outpatient setting (*P* < 0.0001) and noninfectious manifestations (*P* = 0.003). Major infections (defined as requiring intravenous antibiotics or inpatient admission) was the factor second most frequently selected as leading to CGD diagnosis, with 70% of respondents agreeing that this factor almost always (39%; n=14) or often (31%; n=11) leads to CGD identification. Family history was ranked as the third most frequent, with ≈60% of respondents agreeing that this factor almost always (31%; n=11) or often (28%; n=10) leads to CGD identification. Both major infections and family history were ranked as significantly more frequently contributory to CGD diagnosis than were minor infections (both *P* < 0.0001). No respondents indicated that noninfectious CGD manifestations or minor infections were almost always likely to lead to a CGD diagnosis. Less than one-third of respondents reported that these factors were often likely to result in a CGD diagnosis (36% [n=13] for noninfectious manifestations and 14% [n=5] for minor infections).

**Figure 1:**
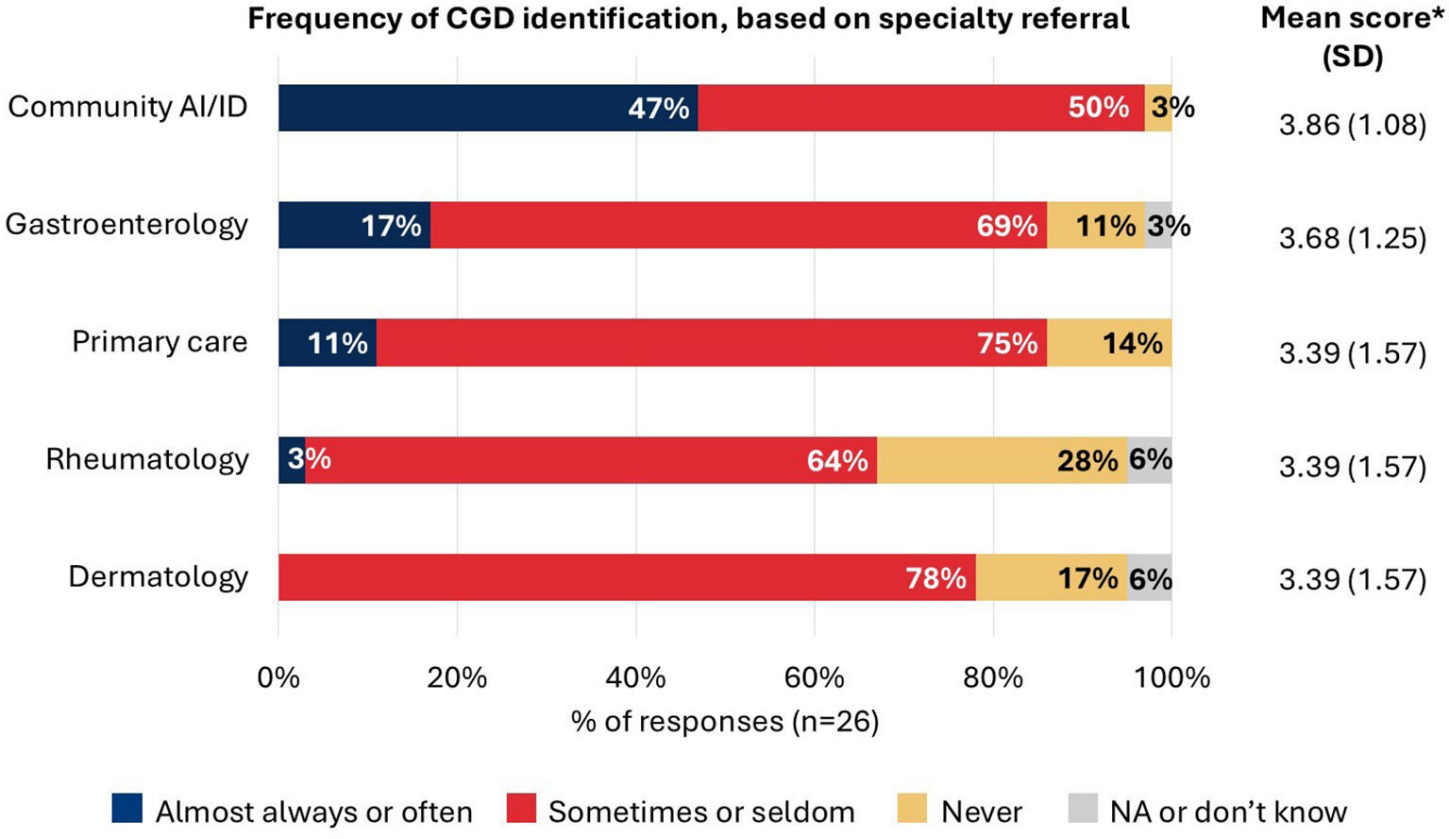
Frequency of CGD identification, based on specialty referral. In your experience, how frequently is CGD identified, based on referrals from these specialties?.*From a 6-point rating scale: 1=Never, 2=Seldom, 3=Sometimes, 4=Often, 5=Almost always, 6=NA or don’t know. AI/ID, allergy-immunology/infectious disease; CGD, chronic granulomatous disease; NA, not applicable.

Most respondents perceived that referrals from medical specialists only occasionally or rarely result in a CGD diagnosis; however, when a diagnosis does occur through a referral, the referral is statistically more likely to have come from a community allergy-immunology/infectious disease specialist vs. a rheumatologist (*P* < 0.0001), dermatologist (*P* < 0.0001), or primary care provider (*P* = 0.037) (**Figure 1**). Other potential referring specialists reported were pulmonologists (n=3) and hematologists (n=2).

A subset of survey items requested clinicians’ perspectives on conceptual statements related to CGD screening. 81% strongly agreed and 19% agreed with ordering CGD genetic testing for individuals with suspected CGD who have low %DHR+. 78% strongly agreed and 19% agreed with CGD screening for at-risk male relatives of patients with XL-CGD, and 67% strongly agreed and 30% agreed with CGD screening for all female relatives of patients with XL-CGD. 41% strongly agreed and 22% agreed with ordering CGD genetic testing for individuals with suspected CGD, independent of %DHR+ results.

The survey also asked respondents to select their top 3 barriers to CGD identification from a provided list. Approximately 75% of respondents selected either delayed recognition of CGD signs/symptoms among health-care providers or the lack of CGD awareness among referring providers as the top barrier limiting CGD identification. A quarter of respondents selected limited awareness about the varying forms of CGD as a top diagnostic barrier. Only 14% selected difficulty obtaining CGD screening and 3% selected family hesitance toward CGD screening as significant barriers to diagnosis.

### 3.3 CGD manifestations

Survey respondents were asked to rank the frequency of several potential CGD manifestations (i.e., major infections, minor infections, presence of CGD-associated pathogens, %DHR+ <20%, absent neutrophil function on DHR testing, and noninfectious inflammatory manifestations) for male patients with XL-CGD, patients with AR-CGD, and symptomatic female carriers of XL-CGD. The presence of CGD-associated pathogens was ranked as 1 of the top 3 CGD manifestations across all CGD subtypes.

Respondents ranked absent neutrophil function as the most frequent CGD characteristic in male patients with XL-CGD and major infections as the most frequent manifestation in patients with AR-CGD. In symptomatic female carriers of XL-CGD, presence of noninfectious inflammatory manifestations was indicated as the most frequent CGD manifestation compared with absent neutrophil function (*P* < 0.0001), major infections (*P* < 0.0001), presence of CGD-associated pathogens (*P* = 0.022), and %DHR+ <20% (*P* = 0.002). 88% of respondents ranked lungs or lymph nodes as the most frequent infection sites, which were statistically significant (both *P* < 0.0001) vs. skin/soft tissue, liver, and bone.

### 3.4 CGD monitoring practices

**Figure 2:**
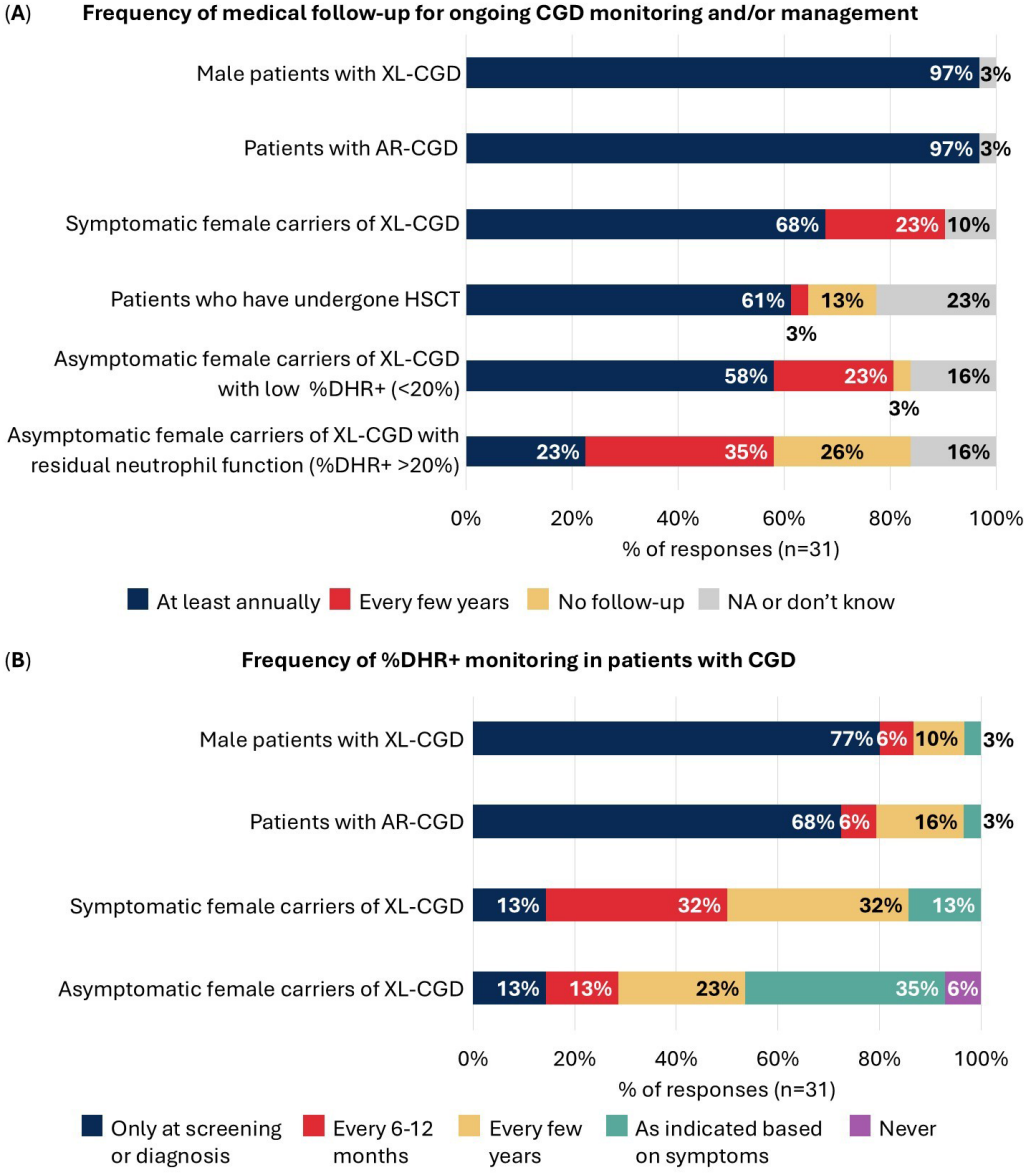
**(A)** Frequency of medical follow-up for ongoing CGD monitoring and/or management. Following CGD diagnosis and treatment initiation, how frequently are these patient groups seen in your practice for ongoing monitoring and/or management?. **(B)** Frequency of %DHR monitoring in patients with CGD. How frequently do you check or monitor %DHR+ in each of these patient groups?. %DHR+, relative proportion of dihydrorhodamine fluorescence–positive neutrophils; AR-CGD, autosomal recessive chronic granulomatous disease; CGD, chronic granulomatous disease; HSCT, hematopoietic stem cell transplantation; NA, not applicable; XL-CGD, X-linked chronic granulomatous disease.

Survey respondents were asked to describe the frequency of clinical touchpoints for male patients with XL-CGD, patients with AR-CGD, symptomatic female carriers of XL-CGD, and asymptomatic female carriers of XL-CGD. Most respondents (97%) reported seeing male patients with XL-CGD and patients with AR-CGD for clinic visits at least annually (**Figure 2A**) and approximately two-thirds (68%) reported annual clinic visits for symptomatic female carriers of XL-CGD. Respondents indicated more frequent clinical touchpoints for asymptomatic female carriers with low %DHR+ compared to carriers with normal neutrophil function. Respondents also reported seeing male patients with XL-CGD and patients with AR-CGD more often than asymptomatic carriers of XL-CGD with %DHR+ <20% (*P* < 0.0001).

There was no significant difference in %DHR+ monitoring approaches for males with XL-CGD and patients with AR-CGD (*P* = 0.949), with most respondents performing %DHR+ testing only at diagnosis for male patients with XL-CGD (77%) and patients with AR-CGD (68%). This contrasted with %DHR+ testing practices for symptomatic and asymptomatic carriers of XL-CGD, for whom testing frequency varies (i.e., typically performed every few months to years or as indicated based on symptoms) (**Figure 2B**).

### 3.5 Considerations influencing CGD treatment recommendations

**Figure 3:**
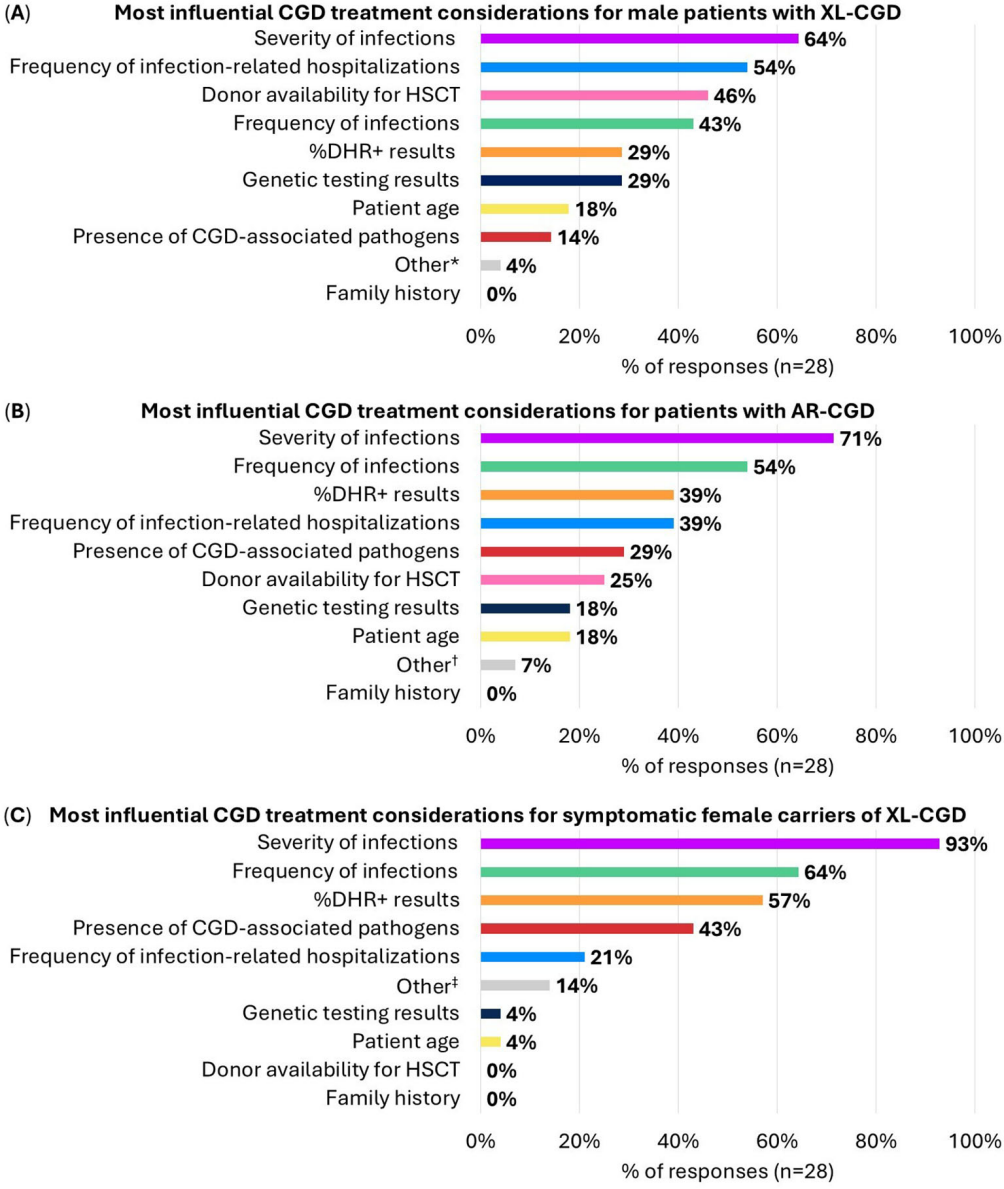
Most influential CGD treatment considerations for **(A)** Male patients with XL-CGD; **(B)** Patients with AR-CGD; **(C)** Symptomatic female carriers of XL-CGD. Based on your experience, please select the top 3 considerations that have the greatest influence on your CGD treatment recommendations for each subtype, with #1 being the most influential. *Responses included other comorbidities and age at diagnosis (n=1) and end organ damage (n=1). ^†^Responses included noninfectious complications (n=2) and presence of autoinflammatory disease (n=1). ^‡^Responses included noninfectious complications (n=2) and other comorbidities (n=1). One respondent indicated they typically choose conservative management. One respondent does not have any patients who are symptomatic female carriers of XL-CGD. %DHR+, relative proportion of dihydrorhodamine fluorescence–positive neutrophils; AR-CGD, autosomal recessive chronic granulomatous disease; CGD, chronic granulomatous disease; HSCT, hematopoietic stem cell transplantation; XL-CGD, X-linked chronic granulomatous disease.

Respondents were asked to rank the influence of potential clinical considerations on their CGD treatment recommendations for male patients with XL-CGD, patients with AR-CGD, and symptomatic female carriers of XL-CGD (**Figures 3A-C**). Infection severity was the highest ranked consideration influencing CGD treatment recommendations across all subtypes. Nearly half (46%) of respondents ranked HSCT donor availability as 1 of the top 3 considerations influencing treatment decisions for male patients with XL-CGD. %DHR results were reported to be a dominant consideration in treatment decisions for symptomatic female carriers of XL-CGD, with 57% of respondents ranking this as 1 of the top 3 considerations influencing treatment decisions for this patient subgroup. Family history was not selected as a top consideration influencing treatment decisions for any patient subgroup.

### 3.6 CGD treatment recommendations

Several survey questions asked respondents to rank the frequency of treatment recommendations (e.g., prophylaxis, HSCT, experimental therapies) for male patients with XL-CGD, patients with AR-CGD, and symptomatic female carriers of XL-CGD. Antibiotic prophylaxis, antifungal prophylaxis, avoidance of environmental triggers, and specialty referrals were identified as the most frequent recommendations across all CGD subtypes. The most frequently recommended CGD treatments for male patients with XL-CGD and patients with AR-CGD were reported, equally, as antibiotic and antifungal prophylaxis, while the top recommended treatments for symptomatic female carriers of XL-CGD were, equally, antibiotic prophylaxis and specialty referrals for noninfectious manifestations. Approximately half (48%) of respondents strongly agreed that additional CGD management practice guidelines would be beneficial.

#### 3.6.1 Prophylaxis recommendations

**Figure 4:**
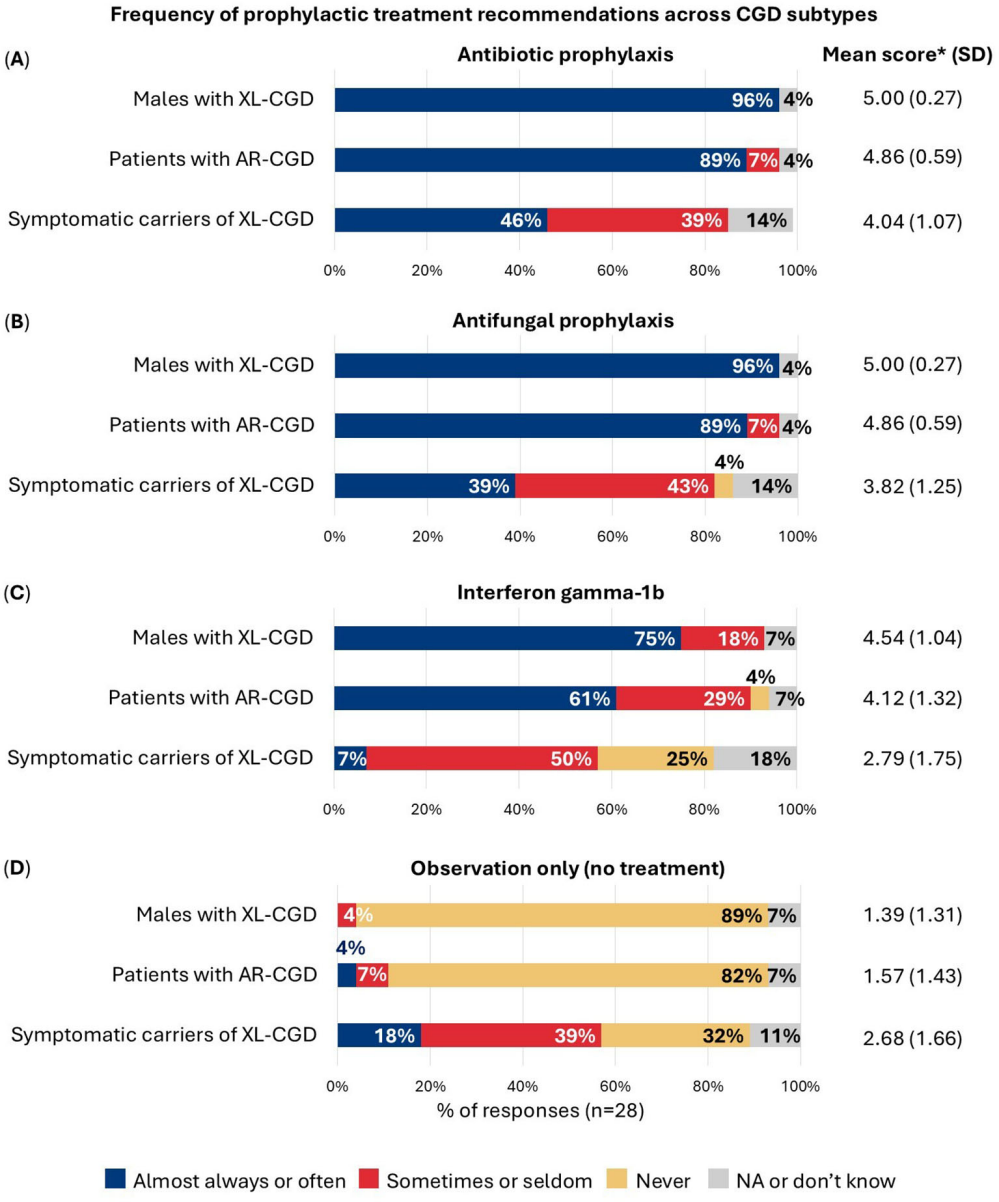
Frequency of prophylactic treatment recommendations across CGD subtypes: **(A)** Antibiotic prophylaxis; **(B)** Antifungal prophylaxis; **(C)** Interferon gamma-1b; **(D)** Observation only (no treatment). How frequently do you recommend these management options for male patients with XL-CGD, patients with AR-CGD, and symptomatic female carriers of XL-CGD? *From a 6-point rating scale: 1=Never, 2=Seldom, 3=Sometimes, 4=Often, 5=Almost always, 6=NA or don’t know. AR-CGD, autosomal recessive chronic granulomatous disease; CGD, chronic granulomatous disease; NA, not applicable; XL-CGD, X-linked chronic granulomatous disease.

Respondents were statistically significantly more likely to report recommending any prophylactic and immunomodulatory treatment (antibiotics, antifungals, or interferon gamma-1b) to male patients with XL-CGD and patients with AR-CGD vs. to symptomatic female carriers of XL-CGD (both *P* < 0.0001). Most respondents indicated that antibiotic prophylaxis and antifungal prophylaxis are almost always or often recommended for male patients with XL-CGD (96%) and patients with AR-CGD (89%), whereas these recommendations for symptomatic female carriers of XL-CGD are less common (**Figures 4A, B**).

75% and 61% of respondents indicated they almost always or often recommend interferon gamma-1b for male patients with XL-CGD and patients with AR-CGD, respectively (**Figure 4C**). This contrasted with symptomatic female carriers of XL-CGD, for whom only 7% of respondents indicated they almost always or often recommend interferon gamma-1b. Although the difference in frequency of interferon gamma-1b recommendations for male patients with XL-CGD and patients with AR-CGD was not significant, the difference in frequency of recommendations was significant for male patients with XL-CGD and patients with AR-CGD compared with symptomatic female carriers of XL-CGD (both *P* < 0.0001).

Most respondents indicated they never recommend observation without treatment for male patients with XL-CGD (89%) and patients with AR-CGD (82%), whereas 18% almost always or often pursue this option for symptomatic female carriers of XL-CGD (**Figure 4D**).

#### 3.6.2 Treatment of granulomas

**Figure 5:**
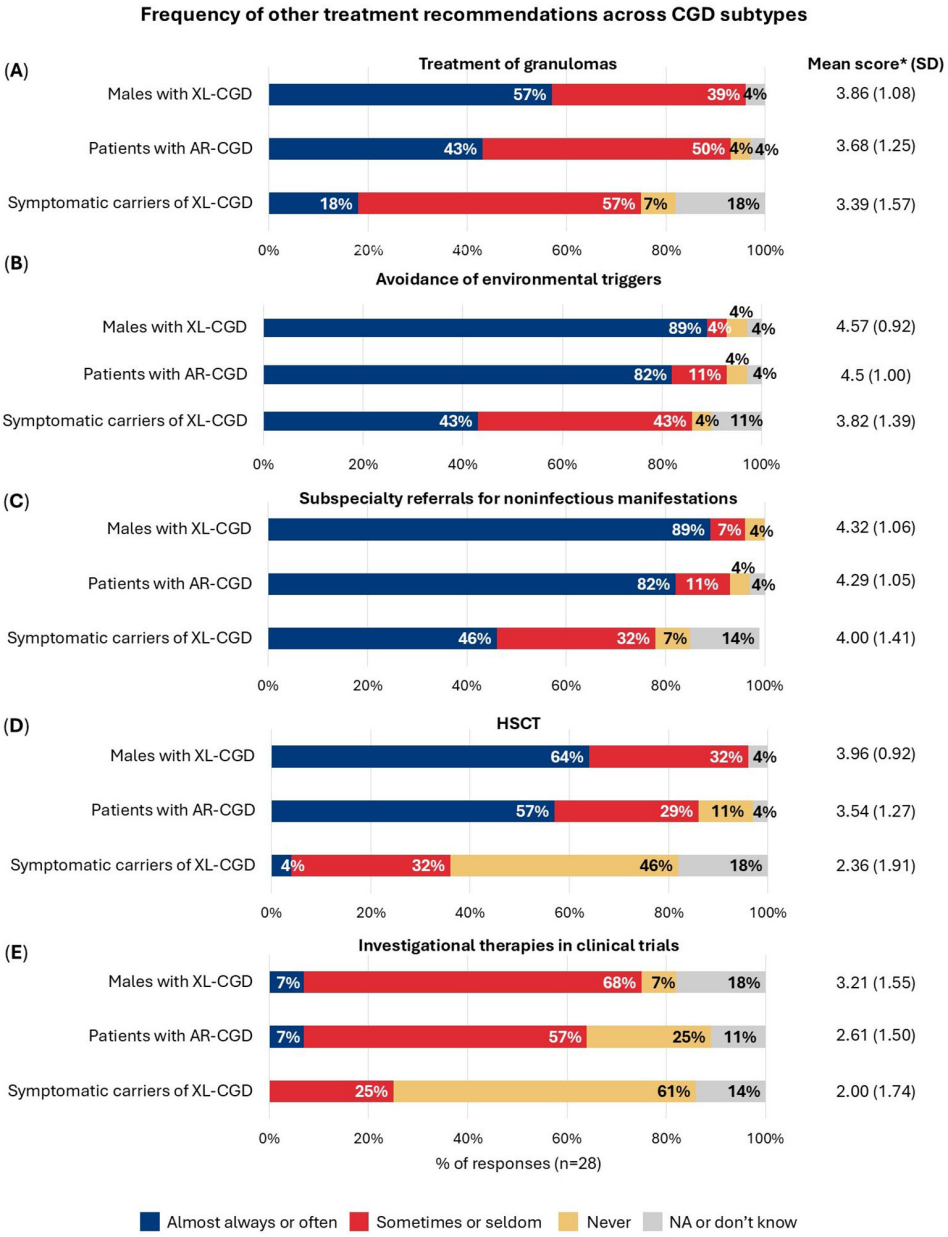
Frequency of other treatment recommendations across CGD subtypes: **(A)** Treatment of granulomas; **(B)** Avoidance of environmental triggers; C) Specialty referrals for noninfectious manifestations; **(D)** HSCT; **(E)** Experimental therapies in clinical trials. How frequently do you recommend these management options for male patients with XL-CGD, patients with AR-CGD, and symptomatic female carriers of XL-CGD?. *From a 6-point rating scale: 1=Never, 2=Seldom, 3=Sometimes, 4=Often, 5=Almost always, 6=NA or don’t know. AR-CGD, autosomal recessive chronic granulomatous disease; CGD, chronic granulomatous disease; HSCT, hematopoietic stem cell transplantation; NA, not applicable; XL-CGD, X-linked chronic granulomatous disease.

Most respondents indicated that they almost always or often recommend treatment of granulomas in male patients with XL-CGD (57%); conversely, most respondents only sometimes or seldom recommend similar treatment for patients with AR-CGD (50%) and symptomatic female carriers of XL-CGD (57%) (**Figure 5A**).

#### 3.6.3 Avoidance of environmental triggers

Most respondents indicated that they almost always or often recommend avoidance of environmental triggers for male patients with XL-CGD (89%) and patients with AR-CGD (82%); however, less than half (43%) similarly recommend this approach for symptomatic female carriers of XL-CGD (**Figure 5B**).

#### 3.6.4 Specialty referrals for noninfectious manifestations

Most respondents indicated they almost always or often recommend specialty referrals for noninfectious manifestations for male patients with XL-CGD (89%) and patients with AR-CGD (82%). Less than half (46%) have the same recommendation for symptomatic female carriers of XL-CGD (**Figure 5C**), despite noninfectious manifestations being ranked as the top CGD manifestation in this patient subgroup.

#### 3.6.5 HSCT recommendations

64% and 57% of respondents almost always or often recommend HSCT for male patients with XL-CGD and patients with AR-CGD, respectively, while only 4% similarly pursue HSCT for symptomatic female carriers of XL-CGD (**Figure 5D**). Respondents were significantly more likely to report recommending HSCT for male patients with XL-CGD vs. symptomatic female carriers of XL-CGD (*P* = 0.008). There was no significant difference in respondents’ likelihood of recommending HSCT for male patients with XL-CGD vs. patients with AR-CGD or for patients with AR-CGD vs. symptomatic female carriers of XL-CGD (both *P* = 1.000).

#### 3.6.6 Investigational therapies

Most respondents indicated they sometimes or seldom recommend investigational therapies (e.g., gene therapy) for male patients with XL-CGD (68%) and patients with AR-CGD (57%), suggesting some willingness to explore investigational treatments for these patient subgroups. In contrast, the majority (61%) of respondents indicated they never recommend this option for symptomatic female carriers of XL-CGD (**Figure 5E**), suggesting less openness to this approach for this subgroup.

### 3.7 Conceptual statements related to the recognition and potential care of patients with AR-CGD and of female carriers of XL-CGD

All respondents agreed that patients with AR-CGD require infection prophylaxis and most respondents (94%) agreed that inadequate recognition of AR-CGD delays time to diagnosis and treatment.

Approximately 90% of respondents strongly agreed or agreed that they routinely ask female carriers of XL-CGD about a wide range of possible CGD-related symptoms, that female carriers of XL-CGD are at risk of developing these manifestations over time, and that they counsel asymptomatic female carriers of XL-CGD to follow up with their health-care providers if they develop any CGD manifestations. 56% strongly agreed and 41% agreed that symptomatic female carriers of XL-CGD may exhibit manifestations warranting treatment with prophylaxis (including antibiotics, antifungals, and/or interferon gamma-1b).

### 3.8 Barriers to CGD care

More than half of respondents ranked treatment of comorbid conditions (61%) and poor adherence to CGD treatment (58%) as the top barriers to CGD management, followed by insurance coverage and/or out-of-pocket costs for CGD treatment (45%), poor access to CGD specialists and medical specialists (42%), and difficulty coordinating multispecialty CGD care (39%). Difficulty administering CGD medications (23%) and side effects of CGD medications (19%) were infrequently selected as top barriers to management. Other potential barriers suggested by respondents included rarity of CGD, disease-specific complications, social issues, the need for repeat monitoring, and refractory complications.

Respondents were also asked to consider the impact of several patient/caregiver SDOH factors on a clinician’s ability to provide effective CGD care. More than two-thirds of respondents consider a patient’s socioeconomic status (74%), physical access to health care (74%), financial status (74%), access to transportation (63%), and health literacy (63%) to have a strong impact on the clinician’s ability to effectively treat patients with CGD. Approximately two-thirds of respondents routinely assess for SDOH barriers to care in their patients with CGD, 56% have access to a social worker who can support their patients as needed, and 32% have access to a dedicated social worker as part of their team. Furthermore, 52% assess mental health in their patients with CGD and refer them to specialized support as needed.

## 4 Discussion

This multidisciplinary survey highlights the perspectives of US-based clinicians with experience in current CGD diagnostic, treatment, and monitoring practices. The results highlight several potential areas of discord related to specialty referrals for CGD diagnosis and management of symptomatic female carriers of XL-CGD.

High clinical suspicion of CGD remains a cornerstone of prompt treatment and improved outcomes. An important finding of this study is that specialty medical referrals do not often lead to CGD diagnosis. This is concerning, as noninfectious manifestations are prevalent in CGD and may be the presenting factor. For example, 50% of patients with CGD are diagnosed with inflammatory bowel disease at some point in their disease course, including some who present with this feature (especially early-onset colitis) as their initial CGD manifestation (21). Noninfectious findings may also be difficult to identify as CGD due to symptom overlap with conditions found in the general population; for example, CGD-related dermatologic findings, such as severe acne, may not be attributed to CGD unless they present with greater severity, recurrence, or become treatment resistant. Additionally, cutaneous lupus–like conditions may similarly be overlooked in patients with CGD, who may not have typical lupus serologic findings (17). Case reports have described the diagnosis of CGD following liver abscess, chorioretinitis, obstructive uropathy, and nonspecific gastrointestinal and other symptoms (22–26). This means that the pool of potential referring providers is quite large, with gastroenterologists, primary care providers, rheumatologists, dermatologists, infectious disease specialists, pulmonologists, and hematologists as potential referring specialists. Greater cross-disciplinary education and collaboration, led by expert clinicians who treat patients with CGD, would be beneficial to help boost awareness of this rare condition among other medical specialists.

Early diagnosis of CGD is important for prompt initiation of CGD treatment, identification of potentially affected family members, and improved clinical outcomes (10, 27). A majority of responding clinicians agreed that the presence of CGD-associated pathogens leads to identification of CGD across all patient subtypes, corresponding with previous reports (7, 28, 29). Infection-related factors (i.e., absent neutrophil function and presence of major infections) were ranked as the most frequent manifestations in male patients with XL-CGD and patients with AR-CGD. In contrast, though not unexpected, noninfectious inflammatory findings were ranked as the most frequent manifestation in symptomatic female carriers of XL-CGD. The clinical burden of autoimmune and/or inflammatory findings has been described in this population (2, 8, 30). These symptoms do not correlate with superoxide production; therefore, %DHR+ testing is not adequate to predict a risk for inflammatory manifestations in female carriers of XL-CGD (8); however, monitoring %DHR+ over time is recommended in female carriers of XL-CGD, as skewed lyonization may change over time, resulting in a change in the need for infection prophylaxis (8). Though there are no standard recommendations for frequency of %DHR+ testing in this population, a recent case series showed %DHR+ changed from ≈20% to ≈7% over ≈1 to 5 years in 2 female carriers of XL-CGD (9), reinforcing the need for ongoing monitoring.

There was more variability reported in seeking family testing for female carriers of XL-CGD vs. male patients with XL-CGD and patients with AR-CGD. Most respondents order CGD screening for at-risk relatives of male patients with XL-CGD, although there is a stronger propensity to do so in male vs. female relatives, even though most respondents recognize that female carriers of XL-CGD may be symptomatic or develop symptoms over time. Encouragingly, nearly all respondents reported that they investigate female carriers of XL-CGD for a wide range of CGD-related symptoms and that they also routinely counsel asymptomatic female carriers to follow up with their practice if they develop new potential CGD manifestations. The ability to counsel female carriers, however, is predicated on these asymptomatic, at-risk female relatives having been screened, diagnosed, and in care. Recently, experts have advocated for CGD screening of all direct female relatives of male patients with XL-CGD upon identification of the proband male patient with XL-CGD (9).

Regarding CGD management, infection severity was the top factor influencing CGD treatment recommendations across all CGD subtypes. Greater alignment in management approach was observed for treatment of male patients with XL-CGD and patients with AR-CGD than for treatment of symptomatic female carriers of XL-CGD. The greater disparity in treatment preferences for symptomatic female carriers of XL-CGD may reflect patients’ symptomatic variability and/or the relative lack of clinical guidance, such as how frequently to see them in follow-up, available for this patient cohort. While most respondents recognized that female carriers of XL-CGD may have infections warranting prophylactic treatment, less than half would recommend antibiotic or antifungal prophylaxis and far fewer have similar convictions about use of interferon gamma-1b in this population. Recent reports describing the management of symptomatic carriers of XL-CGD highlight substantial variation in treatment and monitoring approaches, while also emphasizing the need for comprehensive longitudinal care and further consensus on clinical care strategies (31–33). Taken together with nearly half of survey respondents self-reporting strong support for updating CGD clinical guidelines, the observed heterogeneity in care for symptomatic female carriers of XL-CGD suggests a need for future expert consensus. In the absence of such guidelines, the authors suggest that all carriers be evaluated by a CGD specialist, have DHR testing performed to assess risk of infection, and be counseled on the potential for developing autoimmunity and/or autoinflammation. Some carriers will warrant antimicrobial prophylaxis, immunomodulators, and serial DHR testing.

Mental health and psychological assessment, in addition to typical CGD management, has been recommended to help relieve the burden of this disease (34). In this survey, only half of participants assess mental health in their patients with CGD and refer them to specialized support as indicated. Several studies in the past decades have highlighted that patients with CGD, particularly those on prophylactic therapy vs. HSCT, may face lifelong challenges, such as infections and steroid-dependent autoinflammation, which interrupt daily activities and may contribute to an unfavorable psychosocial prognosis and lower quality of life (35, 36). A study in France highlighted that only half of patients with CGD had been able to follow a pattern of normal schooling as a result of chronic complications and repeated hospitalizations (37). Furthermore, in a separate survey of 171 female carriers of XL-CGD, 91% self-reported mental health symptoms such as anxiety, depression, and low self-esteem related to their carrier status (19). The American Academy of Allergy, Asthma, & Immunology recently advocated for physicians to become more involved in addressing health disparities among patients with primary immune deficiencies such as CGD. This includes performing nonbiased SDOH screening during office visits and providing resources to address SDOH-related barriers, as relevant (38). Although there has been no prior research on the impact of SDOH on CGD care, most survey respondents reported that they currently assess patients/families for SDOH barriers and recognize that factors such as socioeconomic status, physical and financial access to health care, transportation access, and health literacy can have a strong impact on CGD care. Qualitative patient-centered research in this area may be helpful for highlighting how SDOH interact with CGD patient care.

This study has several limitations. The study results are based on a clinician survey and therefore represent subjective, opinion-based perceptions rather than objective clinical outcomes. The survey results should be interpreted with caution when considering best practices related to optimal patient outcomes, as the survey did not evaluate the effectiveness of the clinical decisions described. The relatively small, heterogenous survey sample and relatively low response rate to this voluntary survey may constrain the broader applicability of the findings. Despite the majority of respondents having ≥21 years of medical experience, most reported having cared for <10 patients with each CGD subtype (i.e., male patients with XL-CGD, patients with AR-CGD, symptomatic female carriers of XL-CGD, and asymptomatic female carriers of XL-CGD with low %DHR+) over the course of their career, emphasizing the relative rarity of this disease and the potentially low number of clinicians with experience managing these patients. The survey sample was also predominantly composed of allergy/immunology clinicians, which is consistent with the central role this specialty plays in CGD diagnosis and ongoing management; however, other specialties, including hematology/oncology and infectious disease, are also involved in CGD care (3). Accordingly, the survey findings are likely most reflective of CGD treatment norms and opinions within the allergy/immunology specialty and may not fully capture other multidisciplinary perspectives. Specialty-related bias may be apparent; for example, the observation that community allergy-immunology/infectious disease specialists were most likely to refer patients for CGD workup may reflect the high proportion of allergy/immunology clinicians participating in the survey (≈60% of all respondents). A lack of responses from other specialties (eg, gastroenterology and pulmonology) contributes to gaps in understanding about why referrals from these specialists may potentially be low. The low response rate, although typical for voluntary surveys, introduces uncertainty regarding the extent to which all relevant perspectives are represented and raises the possibility of nonresponse bias. Finally, although the survey instrument was developed by experienced CGD physicians, it was not formally validated prior to deployment given the exploratory nature of the study. As a result, statistical analyses were limited to descriptive and rank-based methods, and do not represent definitive evidence of optimal care.

This survey was intentionally designed to capture perspectives among US-based clinicians and therefore does not provide insight into CGD care practices outside the US. Differences in CGD phenotype and presentation have been well documented across geographical regions, as have differences in clinical management approaches (39). While direct comparisons are challenging given the stratification of this survey’s data by CGD subtype, European registry studies report lower utilization of antibiotics, antifungals, and interferon gamma-1b; higher rates of HSCT; more frequent clinical follow-up; and differences in CGD-related pathogens and availability of diagnostic technologies (1, 5, 35, 40–42). Beyond Europe, cohort studies from Latin America, the Middle East, India, and North Africa similarly describe variations in medical management, lower HSCT utilization, higher rates of mycobacterial disease (particularly *Bacillus Calmette-Guerin*–related complications), differences in common pathogens, variations in diagnostic age, and more limited access to specialized diagnostics, longitudinal care, and treatment options compared to the US (39, 43–47). Across many non-US regions, AR-CGD is also more frequently reported, likely reflecting regional differences in population genetics, founder effects, and consanguinity (5, 47–49). Given these substantial epidemiologic, health-system, and practice-level differences, the findings of this survey cannot be generalized to CGD care outside the US.

To our knowledge, this survey is the first investigation of clinician perceptions of CGD practice trends in the US. Prior CGD-related surveys have focused on patient-reported experiences (19, 36, 50–52). As such, the findings from this survey complement existing patient and family perspectives by providing insight into clinician-reported practices. It is also among the first surveys to include symptomatic carriers of XL-CGD as a distinct patient group warranting specialized consideration. Future investigations could build on this work by seeking opinions from a broader range of geographical areas as well as specialists and by continuing to target infectious disease experts and hematology/oncology specialists, who also did not participate in this survey in large numbers. This survey also showed several areas of high consensus, such as in treatment decisions for male patients with XL-CGD and patients with AR-CGD. Focusing future survey efforts on particularly challenging areas of care (i.e., management of symptomatic female carriers of XL-CGD) may be helpful.

## 5 Conclusion

In summary, this survey highlights the importance of pathogen detection in CGD identification, differences in diagnostic practices and treatment decisions among US-based clinicians, and a need for greater education among specialist clinicians to increase early detection of CGD. The findings also identify several areas of knowledge gaps and clinical discordance in the management of symptomatic female carriers of XL-CGD, especially compared with males with XL-CGD and patients with AR-CGD, for whom clinical decision-making appears more well defined in US-based practice settings. The observed variability in reported clinical practices, particularly with respect to the monitoring and management of symptomatic female carriers of XL-CGD, suggests that additional guidance in this area would be beneficial. Future efforts to engage larger, more diverse clinician populations and incorporate international perspectives should help to better characterize current CGD care practices.

## Data Availability

The raw data supporting the conclusions of this article will be made available by the authors, without undue reservation.
