# Supplementary material for: Perspectives on chronic granulomatous disease: results of a clinician survey
Source: Front Immunol. 2026 Jul 16;17:1731304. doi: 10.3389/fimmu.2026.1731304 (PMC13421416; doi:10.3389/fimmu.2026.1731304)
Supplement: Supplementary file 1 [file DataSheet1.pdf]

Clinician Perspectives on Chronic Granulomatous Disease (CGD) Management Survey

Section 1: Respondent Screening

1. Have you ever been involved in the care of any patients with chronic granulomatous disease (CGD)?
   - o Yes
   - o No [Survey terminates at next page]

2. Where is your practice located?
   - o In the United States
   - o Outside of the United States [Survey terminates at next page]

Section 2: Diagnosis and Patient Journey

3. In your experience, how frequently is CGD identified based on the following factors?

	Never	Seldom	Sometimes	Often	Almost always	NA or don't know
Major infections (ie, requiring IV antibiotics or inpatient admission)	o	o	o	o	o	o
Minor infections (ie, treated outpatient)	o	o	o	o	o	o
Presence of pathogens characteristic of CGD (eg, *Staphylococcus aureus, Serratia marcescens, Burkholderia cepacia*, *Nocardia* species, *Aspergillus* species, *Candida*)	o	o	o	o	o	o
Noninfectious inflammatory manifestations (eg, colitis, inflammatory bowel disease, acne, cutaneous lupus)	o	o	o	o	o	o
Family history of CGD	o	o	o	o	o	o

4. In your experience, how frequently is CGD identified based on referrals from the following subspecialities:

	Never	Seldom	Sometimes	Often	Almost always	NA or don't know
Primary care	o	o	o	o	o	o
Gastroenterology	o	o	o	o	o	o
Dermatology	o	o	o	o	o	o
Rheumatology	o	o	o	o	o	o
Community AI/ID	o	o	o	o	o	o
Other subspecialties; please specify below	o	o	o	o	o	o

5. From the list below, please select the **top 2** barriers limiting prompt identification of patients with CGD, with #1 being the greatest barrier.

Delayed recognition of CGD signs and symptoms among clinicians
Need for greater awareness of the varying infectious and noninfectious presentations of CGD among potential referring clinicians
Need for greater awareness among potential CGD treaters of varying forms of CGD (eg, autosomal recessive CGD [AR-CGD] or symptomatic female carriers of X-linked CGD [XL-CGD])
Difficulty obtaining CGD screening (eg, dihydrorhodamine [DHR] testing)
Family hesitancy to move forward with CGD testing
Other; please specify: __________________________ [70 characters max]

Section 3: CGD Symptoms and Infections

6. Please rank the manifestations listed below in order of frequency (#1 being most often, #6 being least often) for **males with XL-CGD**.

Major infections (ie, requiring IV antibiotics or inpatient admission)
Minor infections (ie, treated outpatient)
Presence of pathogens characteristic of CGD (eg, *Staphylococcus aureus, Serratia marcescens, Burkholderia cepacia*, *Nocardia* species, *Aspergillus* species, *Candida*)
%DHR+ <20%
Absent neutrophil function on DHR testing
Noninfectious inflammatory manifestations (eg, colitis, inflammatory bowel disease, acne, cutaneous lupus)

7. Please rank the manifestations listed below in order of frequency (#1 being most often, #6 being least often) for **patients with AR-CGD**.

Major infections (ie, requiring IV antibiotics or inpatient admission)
Minor infections (ie, treated outpatient)
Presence of pathogens characteristic of CGD (eg, *Staphylococcus aureus, Serratia marcescens, Burkholderia cepacia*, *Nocardia* species, *Aspergillus* species, *Candida*)
%DHR+ <20%
Absent neutrophil function on DHR testing
Noninfectious inflammatory manifestations (eg, colitis, inflammatory bowel disease, acne, cutaneous lupus)

8. Please rank the manifestations listed below in order of frequency (#1 being most often, #6 being least often) for **symptomatic female carriers of XL-CGD**.

Major infections (ie, requiring IV antibiotics or inpatient admission)
Minor infections (ie, treated outpatient)
Presence of pathogens characteristic of CGD (eg, *Staphylococcus aureus, Serratia marcescens, Burkholderia cepacia*, *Nocardia* species, *Aspergillus* species, *Candida*)
%DHR+ <20%
Absent neutrophil function on DHR testing
Noninfectious inflammatory manifestations (eg, colitis, inflammatory bowel disease, acne, cutaneous lupus)

9. Based on your experience, please select the **top 3** most frequently occurring sites of infection in patients with CGD.
   - o Lungs
   - o Lymph nodes
   - o Liver
   - o Bone
   - o Skin/soft tissue
   - o Other; please specify

Section 4: CGD Management

10. Which of the below team members provide care to patients with CGD at your practice? Select all that apply.
    - o Physician
    - o Physician fellow or trainee
    - o Advanced practice provider
    - o Registered nurse
    - o Genetic counselor
    - o Social worker
    - o Clinical coordinator
    - o Other; please specify

11. Following CGD diagnosis and treatment initiation, how frequently are the below patient groups seen in your practice for ongoing monitoring and/or management?

	**≥2x/year**	**Annually**	**Every few years**	**No follow-up**	**NA or don't know**
Males with XL-CGD	o	o	o	o	o
Patients with AR-CGD	o	o	o	o	o
Symptomatic female carriers of XL-CGD	o	o	o	o	o
Asymptomatic female carriers of XL-CGD with low %DHR+ (<20%)	o	o	o	o	o
Asymptomatic female carriers of XL-CGD with residual neutrophil function (%DHR+ >20%)	o	o	o	o	o
Patients who have undergone HSCT	o	o	o	o	o

12. How frequently do you check or monitor %DHR+ in each of the below patient groups?

	**Only at screening or diagnosis**	**Every 6-12 months**	**Every few years**	**As indicated based on symptoms**	**Never**	**NA or don't know**
Males with XL-CGD	o	o	o	o	o	o
Patients with AR-CGD	o	o	o	o	o	o
Symptomatic female carriers of XL-CGD	o	o	o	o	o	o
Asymptomatic female carriers of XL-CGD	o	o	o	o	o	o

13. From the list below, please select the **top 3** barriers related to CGD management, with #1 being the greatest barrier.

Side effects of CGD medications
Difficulty administering CGD medications
Insurance coverage and/or out of pocket costs for CGD medications
Poor adherence to CGD treatments
Poor access to CGD treaters and other medical subspecialities
Difficulty coordinating CGD care across multiple subspecialties
Management and treatment of comorbidities in addition to typical CGD treatment (eg, IBD)
Other; please specify

14. How frequently do you recommend each of the below management options for **males with XL-CGD**?

	**Never**	**Seldom**	**Sometimes**	**Often**	**Almost always**	**NA or don't know**
Antibiotic prophylaxis	o	o	o	o	o	o
Antifungal prophylaxis	o	o	o	o	o	o
Interferon gamma-1b	o	o	o	o	o	o
Treatment of granulomas (eg, steroids)	o	o	o	o	o	o
Avoidance of environmental triggers (eg, raking leaves, swimming in fresh water or salt water)	o	o	o	o	o	o
Hematopoietic stem cell transplant (HSCT)	o	o	o	o	o	o
Subspecialty referrals for noninfectious manifestations (eg, colitis, inflammatory bowel disease, acne, cutaneous lupus)	o	o	o	o	o	o
Experimental therapies in clinical trials (eg, gene therapy)	o	o	o	o	o	o
Observation only (no treatment)	o	o	o	o	o	o

15. Based on your experience, please select the **top 3** considerations that have the greatest influence on your CGD treatment recommendations for **males with XL-CGD**, with #1 being the most influential.

Frequency of infections
Severity of infections
Frequency of infection-related hospitalizations
Presence of pathogens characteristic of CGD (eg, *Staphylococcus aureus, Serratia marcescens, Burkholderia cepacia, Nocardia* species, *Aspergillus* species, *Candida*)
Genetic testing results
%DHR+ results
Patient age
Family history
Donor availability for HSCT
Other; please specify

16. How frequently do you recommend each of the below management options for **patients with AR-CGD**?

	Never	Seldom	Sometimes	Often	Almost always	NA or don't know
Antibiotic prophylaxis	o	o	o	o	o	o
Antifungal prophylaxis	o	o	o	o	o	o
Interferon gamma-1b	o	o	o	o	o	o
Treatment of granulomas (eg, steroids)	o	o	o	o	o	o
Avoidance of environmental triggers (eg, raking leaves, swimming in fresh water or salt water)	o	o	o	o	o	o
HSCT	o	o	o	o	o	o
Subspecialty referrals for noninfectious manifestations (eg, colitis, inflammatory bowel disease, acne, cutaneous lupus)	o	o	o	o	o	o
Experimental therapies in clinical trials (eg, gene therapy)	o	o	o	o	o	o
Observation only (no treatment)	o	o	o	o	o	o

17. Based on your experience, please select the **top 3** considerations that have the greatest influence on your CGD treatment recommendations for **patients with AR-CGD**, with #1 being the most influential.

Frequency of infections
Severity of infections
Frequency of infection-related hospitalizations
Presence of pathogens characteristic of CGD (eg, *Staphylococcus aureus, Serratia marcescens, Burkholderia cepacia, Nocardia* species, *Aspergillus* species, *Candida*)
Genetic testing results
%DHR+ results
Patient age
Family history
Donor availability for HSCT
Other; please specify

18. How frequently do you recommend each of the below management options for **symptomatic female carriers of XL-CGD**?

	Never	Seldom	Sometimes	Often	Almost always	NA or don't know
Antibiotic prophylaxis	o	o	o	o	o	o
Antifungal prophylaxis	o	o	o	o	o	o
Interferon gamma-1b	o	o	o	o	o	o
Treatment of granulomas (eg, steroids)	o	o	o	o	o	o
Avoidance of environmental triggers (eg, raking leaves, swimming in fresh water or salt water)	o	o	o	o	o	o
HSCT	o	o	o	o	o	o
Subspecialty referrals for noninfectious manifestations (eg, colitis, inflammatory bowel disease, acne, cutaneous lupus)	o	o	o	o	o	o

Experimental therapies in clinical trials (eg, gene therapy)	o	o	o	o	o	o
Observation only (no treatment)	o	o	o	o	o	o

19. Based on your experience, please select the **top 3** considerations that have the greatest influence on your CGD treatment recommendations for **symptomatic female carriers of XL-CGD**, with #1 being the most influential.

Frequency of infections
Severity of infections
Frequency of infection-related hospitalizations
Presence of pathogens characteristic of CGD (eg, *Staphylococcus aureus, Serratia marcescens, Burkholderia cepacia, Nocardia* species, *Aspergillus* species, *Candida*)
Genetic testing results
%DHR+ results
Patient age
Family history
Donor availability for HSCT
Other; please specify

Section 5: Social Determinants of Health in CGD Care

20. Social determinants of health (SDOH) are the conditions in which people are born, grow, work, live, and age. In your experience, how strongly do each of the below patient/caregiver SDOH factors impact your ability to effectively treat patients with CGD?

	No impact	Mild impact	Strong impact	NA or don't know
Socioeconomic status	o	o	o	o
Employment status	o	o	o	o
Food insecurity	o	o	o	o
Housing stability	o	o	o	o
Neighborhood and built environment	o	o	o	o
Access to transportation	o	o	o	o
Physical access to healthcare	o	o	o	o
Financial access to healthcare	o	o	o	o
Health literacy	o	o	o	o
Education level	o	o	o	o
English proficiency	o	o	o	o
Discrimination and/or racism	o	o	o	o

Section 6: CGD Perspectives and Attitudinal Questions

21. Please rate your level of agreement with the following statements.

	Strongly disagree	Disagree	Neutral	Agree	Strongly agree	NA or don't know
I recommend CGD screening for at-risk male relatives of patients with XL-CGD	o	o	o	o	o	o
I recommend CGD screening for all female relatives of patients with XL-CGD	o	o	o	o	o	o
I order CGD genetic testing for individuals with suspected CGD who have low %DHR+	o	o	o	o	o	o
I order CGD genetic testing for individuals with suspected CGD independent of the results of DHR testing	o	o	o	o	o	o
Asymptomatic female carriers of XL-CGD are at risk of CGD-related infections in the long-term due to potential skewed lyonization over time	o	o	o	o	o	o
I routinely counsel asymptomatic female carriers of XL-CGD to follow up in my practice if they develop any CGD manifestations	o	o	o	o	o	o
I routinely ask XL-CGD carriers about a wide range of possible symptoms indicative of disease	o	o	o	o	o	o
Symptomatic female carriers of XL-CGD may exhibit manifestations warranting treatment with prophylaxis (ie, antibiotics, antifungals, and/or interferon gamma-1b)	o	o	o	o	o	o
Patients with AR-CGD may exhibit manifestations warranting treatment with prophylaxis (ie, antibiotics, antifungals, and/or interferon gamma-1b)	o	o	o	o	o	o
Inadequate recognition of AR-CGD delays time to diagnosis and treatment	o	o	o	o	o	o
I routinely assess for SDOH barriers to care in patients with CGD	o	o	o	o	o	o
My practice has access to a social worker who is knowledgeable about CGD and can support patients/families in my clinic as needed	o	o	o	o	o	o
I assess mental health in patients/families with CGD and refer them to specialized support as needed	o	o	o	o	o	o
Clinicians and patients would benefit from additional CGD management practice guidelines	o	o	o	o	o	o

Section 7: Respondent Demographics

22. What is your primary medical specialty?
    - o Allergy/immunology
    - o Infectious disease
    - o Hematology/oncology
    - o Other; please specify

23. How long have you practiced in your current medical specialty?
    - o 0 to 10 years
    - o 11 to 20 years
    - o ≥21 years

24. Which most accurately represents your patient population?
   - Mainly adults (≥18 years old)
   - Mainly pediatrics (≤17 years old)
   - Both adult and pediatric patients

25. Please estimate the number of patients with CGD whom you have cared for.

	**None**	**1 to 10**	**11 to 20**	**≥21**
Males with XL-CGD	o	o	o	o
Patients with AR-CGD	o	o	o	o
Symptomatic female carriers of XL-CGD	o	o	o	o
Asymptomatic female carriers of XL-CGD with low %DHR+	o	o	o	o

Thank you for completing this survey.
